# Comparative metabonomic analysis of hepatotoxicity induced by acetaminophen and its less toxic meta-isomer

**DOI:** 10.1007/s00204-015-1655-x

**Published:** 2016-01-09

**Authors:** Michael Kyriakides, Lea Maitre, Brendan D. Stamper, Isaac Mohar, Terrance J. Kavanagh, John Foster, Ian D. Wilson, Elaine Holmes, Sidney D. Nelson, Muireann Coen

**Affiliations:** 1 Biomolecular Medicine, Division of Computational and Systems Medicine, Department of Surgery and Cancer, Imperial College London, London, SW7 2AZ UK; 2 School of Pharmacy, Pacific University, Hillsboro, OR 97123 USA; 3 Departments of Medicinal Chemistry and Environmental and Occupational Health Sciences, University of Washington, Seattle, WA 98195 USA; 4 ToxPath Sciences Ltd, 1 Troutbeck Avenue, Congleton, Cheshire CW12 4JA UK

**Keywords:** *N*-acetyl-*p*-aminophenol (APAP), *N*-acetyl-*m*-aminophenol (AMAP), Metabonomics/Metabolic Phenotyping, Nuclear magnetic resonance spectroscopy, Hepatotoxicity

## Abstract

The leading cause of drug-induced liver injury in the developed world is overdose with *N*-acetyl-*p*-aminophenol (APAP). A comparative metabonomic approach was applied to the study of both xenobiotic and endogenous metabolic profiles reflective of in vivo exposure to APAP (300 mg/kg) and its structural isomer *N*-acetyl-*m*-aminophenol (AMAP; 300 mg/kg) in C57BL/6J mice, which was anchored with histopathology. Liver and urine samples were collected at 1 h, 3 h and 6 h post-treatment and analyzed by ^1^H nuclear magnetic resonance (NMR) spectroscopy and gas chromatography–mass spectrometry (liver only). Histopathology revealed the presence of centrilobular necrosis from 3 h post-APAP treatment, while an AMAP-mediated necrotic endpoint was not observed within the timescale of this study, yet two of five treated mice showed minimal centrilobular eosinophilia. The ^1^H-NMR xenobiotic metabolic profile of APAP-treated animals comprised of mercapturate (urine and liver) and glutathionyl (liver) conjugates detected at 1 h post-treatment. This finding corroborated the hepatic endogenous metabolic profile which showed depletion of glutathione from 1 h onwards. In contrast, AMAP glutathionyl conjugates were not detected, nor was AMAP-induced depletion of hepatic glutathione observed. APAP administration induced significant endogenous hepatic metabolic perturbations, primarily linked to oxidative and energetic stress, and perturbation of amino acid metabolism. Early depletion of glutathione was followed by depletion of additional sulfur-containing metabolites, while altered levels of mitochondrial and glycolytic metabolites indicated a disruption of energy homeostasis. In contrast, AMAP administration caused minimal, transient, distinct metabolic perturbations and by 6 h the metabolic profiles of AMAP-treated mice were indistinguishable from those of controls.

**Electronic supplementary material:**

The online version of this article (doi:10.1007/s00204-015-1655-x) contains supplementary material, which is available to authorized users.

## Introduction

Paracetamol otherwise known as acetaminophen or *N*-acetyl-*p*-aminophenol (APAP) is a commonly used analgesic and antipyretic drug that can cause extensive liver damage after an excessive dose and is the leading cause of drug-induced liver injury in the USA (Lee 2012). Consequently, it has been extensively studied and is classified as a ‘model hepatotoxin’ given the extensive knowledge of its mechanism of hepatotoxicity (McGill et al. 2012a; Russmann et al. 2009).

APAP predominantly undergoes conjugation via glucuronidation or sulphation in the liver (>90 % of a therapeutic dose). Cytochrome P450 enzymes, principally CYP2E1 (rodents and humans) and CYP3A4 (humans), are responsible for oxidation of APAP to its reactive metabolite, *N*-acetyl-*p*-benzoquinone imine (NAPQI) (Dahlin and Nelson 1982; Miner and Kissinger 1979). NAPQI is a potent oxidant and electrophile, which leads to glutathione depletion, as this represents the primary conjugation and detoxification mechanism, but also protein thiol oxidation, cross-linking and arylation (Bessems and Vermeulen 2001; Miner and Kissinger 1979).

NAPQI has also been shown to covalently bind to mitochondrial proteins (Halmes et al. 1996; Landin et al. 1996), and this is considered to be an important hallmark of APAP-induced hepatotoxicity (Jaeschke et al. 2012). Physiologically, the mitochondria display an altered morphology, including an abrupt increase in volume as a result of mitochondrial membrane transition pore opening (Masubuchi et al. 2005; Pierce et al. 2002). Functionally, there is an overall disruption as reflected by mitochondrial oxidative stress, ATP pool depletion and lower respiration rates (Katyare and Satav 1989; Placke et al. 1987), together with DNA fragmentation mediated by the translocation of nucleases located in the mitochondrial inter-membrane space (Bajt et al. 2006).

*N*-acetyl-*m*-aminophenol (AMAP), a reportedly non-hepatotoxic regioisomer of APAP in mice and hamsters (Nelson 1980; Roberts and Jollow 1978), has been used in comparative studies with APAP (Beyer et al. 2007; Hadi et al. 2013; Howell et al. 2014; Stamper et al. 2010). The comparison of regioisomers with differential toxic liabilities presents significant potential for elucidating mechanisms of toxicity. AMAP is thought to be less hepatotoxic than APAP because it binds to mitochondrial proteins to a lesser extent and primarily binds to cytosolic and microsomal proteins (Streeter et al. 1984; Tirmenstein and Nelson 1989). However, recent evidence suggests that AMAP is toxic in precision-cut liver slices from rats and humans (Hadi et al. 2013), and primary human hepatocytes (Xie et al. 2015), and therefore, further work is required to understand the differential toxicology of the regioisomers across and within species.

Metabonomics is a ‘top-down’ system approach used to describe the metabolic phenotype of biological samples under specific biological conditions or in response to an intervention (Nicholson et al. 1999). Metabonomics (and the related field of metabolomics) has found widespread application in the investigation of molecular toxicology, where the site- and mechanism-specific effects of a toxin or therapeutic intervention can be investigated (Coen 2014; Coen et al. 2008; Lindon et al. 2005). Earlier metabonomic studies have been applied to investigate the metabolism of APAP in both in vivo models and humans (Bales et al. 1984; Nicholls et al. 1995; Spurway et al. 1990). More recently, studies of APAP in mice reported a significant perturbation of metabolites involved in the biosynthesis of glutathione; opthalmate, 5-oxoproline and taurine (Soga et al. 2006; Ghauri et al. 1993), as well as numerous system-level metabolic changes that together suggested a disturbance of energy metabolism, more specifically of increased rates of glycolysis and impaired β-oxidation (Coen et al. 2003, 2004). Further evidence for APAP-induced inhibition of fatty acid β-oxidation includes the observation of elevated levels of serum long-chain acyl-carnitines in mice (Chen et al. 2009) and children (Bhattacharyya et al. 2014).

Here we describe the results of a multiplatform metabonomic study, using gas chromatography–mass spectrometry (GC–MS) and ^1^H nuclear magnetic resonance (NMR) spectroscopy to characterize the system-level xenobiotic metabolic profile together with the hepatic endogenous metabolic consequences of APAP and AMAP administration in mice, with a particular focus on the identification of differential discriminatory metabolites reflective of mitochondrial function and oxidative stress.

## Materials and methods

### Animal handing and treatment

Male C57BL/6J mice (*n* = 50, aged 10 weeks) purchased from Jackson Laboratory (Bar Harbor, ME, USA) were kept in a pathogen-free environment at the University of Washington. The animals were housed in Association for Assessment and Accreditation of Laboratory Animal Care International (AAALAC)-accredited temperature-controlled rooms with a 12-h light/dark cycle throughout the study. They were acclimated to the facility for 1 week during which they had access to ad libitum rodent chow diet and acidified sterile water (pH = 2.77). All experiments were conducted under protocol approved by the Institutional Animal Care and Use Committee of the University of Washington.

After a fasting period of 12 h, the mice were treated with APAP (300 mg/kg in saline; *n* = 15), AMAP (300 mg/kg in saline; *n* = 15) or sterile saline (control; *n* = 20), via I.P. injections of 15 μl/g body weight. The selected APAP dose has been previously reported to cause marked liver damage in mice (Masubuchi et al. 2005; McGill et al. 2012b) and an equimolar dose of AMAP was selected for comparative purposes which has previously been reported to be non-toxic in this strain of mice (Fountoulakis et al. 2000; Priyadarsiny et al. 2008). Food was returned to mice after APAP/AMAP administration.

Mice from each treatment group were euthanized via CO_2_ inhalation and cervical dislocation at 1 h, 3 h and 6 h post-treatment (1 h, 3 h and 6 h, respectively; *n* = 5 for drug treatment groups at each time-point and *n* = 6, *n* = 7 and *n* = 7 for the control groups at 1 h, 3 h and 6 h, respectively). At each time-point, blood from a cardiac puncture was collected into serum separator tubes (Microtainer, BD Biosciences, San Jose, California, USA) together with liver sections from the left lateral lobe, which were immediately snap-frozen in liquid nitrogen and stored at −80 °C. Sera fractions were collected following incubation of the collected blood at room temperature (30 min) and centrifugation (4000×*g* for 6 min). Urine was collected on ice in conical tubes containing sodium azide (1 mL, 1 % w/v water) across the following time periods: 0–1, 1–3 and 3–6 h (*n* = 3 for APAP- and AMAP-treated groups at 1 h and *n* = 5 for all other groups).

### Liver histopathology

Liver tissue sections from the medial lobe were fixed in 10 % paraformaldehyde (formalin) overnight prior to dehydration, paraffin embedding and staining with hematoxylin and eosin (H&E). These were examined using light microscopy, and the extent of centrilobular necrosis was assessed without any prior knowledge of the treatment class. The histopathological findings were carried out for all treated animals and one control per time-point and were graded as recommended in current guidelines for reporting these changes (Ward and Thoolen 2011). The scoring criteria for centrilobular necrosis were as follows: 0 (no lesion), 1 (necrosis of single layer of cells around the central vein affecting <20 % of the central veins in the liver lobes; minimal), 2 (necrosis of single layer of hepatocytes around all of the central veins in the liver lobes; mild) and 3 (necrosis of 2–4 layers of hepatocytes around all of the central veins in most liver lobes; moderate). The histopathological analysis also included assessment and grading of periportal glycogen, focal mixed inflammatory reaction, centrilobular eosinophilia and panlobular fat vacuolation with the analysis for individual animals provided in the Suppl. material (Table 1) together with the grading explanations.

## ^1^H-NMR spectroscopy of the hepatic aqueous soluble component

Liver tissue metabolite extraction for ^1^H-NMR spectroscopic analysis was performed according to the protocol described by Beckonert et al. (2007). Briefly, ice-cold acetonitrile/water (1.5 mL, 1:1) was added to the liver tissue samples (average weight of 41.4 mg and STD ± 3.1 mg. The samples were homogenized with 5 mm stainless steel beads in a homogenizer (Qiagen Tissue Lyser, Retsch GmBH, Haan, Germany) at 25 Hz for 8 min. The samples were then kept on ice for 10 min prior to centrifugation at 17,000×*g* for 15 min at 4 °C (Biofuge Pico, Heraeus, Hanau, Germany). The supernatant was concentrated and dried overnight in a centrifugal evaporator (SpeedVac, Thermoscientific, Waltham, Massachusetts, USA) at 30 °C. The resultant dried supernatant was reconstituted in phosphate buffer (600 μL of a 0.2 M solution containing 99.9 % D_2_O, 3 mM sodium azide (NaN_3_) and 1 mM 3-(trimethylsilyl)-[2,2,3,3-^2^H_4_]-propionic acid sodium salt (TSP)), vortexed for 30 s and then centrifuged at 17,000x*g* for 15 min at 4 °C (Biofuge Pico). The supernatant (550 μL) was placed in 5 mm NMR tubes (outer diameter; NMR Precision tube 507-HP-7, Norell, Landisville, New Jersey, USA). NMR spectral data were acquired on a Bruker Avance-600 spectrometer operating at 600.13 MHz (14.1 T) ^1^H frequency and at a temperature of 300 K using a Bruker TXI probe (Bruker Biospin, Rheinstetten, Germany) and an automated sample handling carousel (Bruker). A standard one-dimensional solvent suppression pulse sequence was used to acquire the free induction decay (FID; relaxation delay—90° pulse—4 μs delay—90° pulse–mixing time–90° pulse–acquire FID) (Beckonert et al. 2007). The D_2_O present in the buffer provided a field frequency lock, whilst the TSP served as the chemical shift reference compound (δ^1^H = 0.00). For each experiment, 256 transients were collected into 64,000 data points using a spectral width of 12,000 Hz, with a relaxation delay of 4 s, mixing time of 100 ms and an acquisition time of 4.5 s.

## ^1^H-NMR spectroscopy of urine

Urine samples were prepared for ^1^H-NMR spectroscopy as previously described (Beckonert et al. 2007). Briefly, urine was mixed with phosphate buffer (2:1, 600 μL total volume; same buffer with the hepatic aqueous extract analysis) and vortexed for 1 min. The samples were then centrifuged at 17,000×*g* for 15 min at 4 °C (Biofuge Pico) and the supernatants (550 μL) transferred to 5 mm NMR tubes (507-HP-7). ^1^H-NMR spectral data were acquired on a Bruker Avance-600 spectrometer as described for the aqueous hepatic extracts.

## ^1^H-NMR spectral data processing

The ^1^H-NMR spectra were initially processed in TopSpin 3.0 NMR Software (Bruker), where a line-broadening factor of 0.3 Hz was applied to all spectra prior to Fourier transformation (FT). The spectra were then manually phased, baseline-corrected and referenced to the TSP peak for the aqueous soluble liver extract and urine spectra or lactate peak for the sera spectra. Full-resolution ^1^H-NMR data were imported into MATLAB (R2012, Mathworks Inc., Natick, Massachusetts, USA), using an in-house script, for further processing, which included the removal of the TSP and water resonance regions before performing probabilistic quotient normalization (Dieterle et al. 2006). This is a robust method of normalization which corrects for the differential dilution of urine samples, a factor which affects the concentration of all metabolites or intensity of all resonances in a spectrum. This dilution factor correction thus enables the detection of the biologically relevant, relative concentration changes in selected metabolites. The method scales the spectra based on the most probable dilution factor, calculated from the distribution of quotients of the intensity of each spectral data point relative to a reference spectrum. This method has shown to be more robust for normalization of metabolic profiling data sets than total area integral normalization (Dieterle et al. 2006). Spectral metabolite assignments were achieved using Statistical TOtal Correlation Spectroscopy (STOCSY) (Cloarec et al. 2005), 2D-NMR experiments (Correlation Spectroscopy), spectral databases (Human Metabolome Database and Biological Magnetic Resonance Bank), software including Chenomx NMR Suite (Chenomx, Edmonton, Alberta, Canada) and previously published assignments (Nicholson et al. 1995). Furthermore, the following hepatic metabolites were identified by ‘spike-in’ experiments with the pure standard compounds: adenosine monophosphate (AMP), succinate, 2-aminoadipate, dimethylamine, phosphocholine, choline and glutathione (reduced and oxidized). A summary of the integral regions of the endogenous metabolites and the drug-related resonances is displayed in Table 2 and Table 3 in the Suppl. material, respectively. Finally, the assignment of APAP and AMAP metabolites was based on existing literature (Bales et al. 1984; Nicholls et al. 2006).

In-house scripts were used to calculate the integral of resonances belonging to drug related, and parent compounds in both treatment groups at 1 h (*n* = 3 for each group), in order to estimate their relative abundance to the parent molecule. The integrated resonances in the hepatic extract ^1^H-NMR profiles were as follows: APAP parent (δ^1^H = 7.26; doublet), APAP glucuronide conjugate (δ^1^H = 7.15; doublet), APAP glutathionyl conjugate (δ^1^H = 6.96; doublet), APAP-*N*-acetylcysteinyl conjugate (δ^1^H = 1.85; singlet), AMAP glucuronide conjugate (δ^1^H = 7.24; singlet) and AMAP parent (δ^1^H = 7.03; singlet). The resonances used in the urine ^1^H-NMR metabolic profiles were as follows: APAP parent (δ^1^H = 6.88; doublet), APAP glucuronide conjugate (δ^1^H = 7.36; doublet), APAP sulfate conjugate (δ^1^H = 7.31; doublet), APAP cysteinyl conjugate (δ^1^H = 7.50; singlet), APAP-*N*-acetylcysteinyl conjugate (δ^1^H = 1.86; singlet), AMAP glucuronide conjugate (δ^1^H = 7.24; singlet), AMAP sulfate conjugate (δ^1^H = 7.43; triplet), AMAP parent (δ^1^H = 7.02; singlet) and APAP methoxy conjugate (δ^1^H = 3.88; singlet). Each integral was adjusted for the equivalent number of protons before the ratio to the parent molecule was calculated. Finally, the integral of the resonances belonging to endogenous metabolites were also calculated, including total glutathione (oxidized and reduced; δ^1^H = 2.55; multiplet), succinate (δ^1^H = 2.41; singlet), 2-aminoadipate (δ^1^H = 2.25; triplet), glutamate (δ^1^H = 2.35; multiplet), d-3-Hydroxybutyrate (d-3-HB; δ^1^H = 1.20; doublet), glucose (δ^1^H = 5.25; doublet), AMP (δ^1^H = 8.61; singlet) and valine (δ^1^H = 1.04; doublet).

### GC–MS analysis of the hepatic aqueous soluble component

Liver tissue samples of an average weight of 25.25 mg and STD of ±0.22 mg were added to an ice-cold HPLC-grade water/methanol mixture (1:1, 1.2 mL total volume) and homogenized with zirconia beads at 6500 Hz (Precellys, Montigny-le-Bretonneux, France) for two 45-s periods with an intermediate 5-min cooling period on dry ice. The homogenized mixtures were incubated for 45 min on ice before centrifugation at 17,000×*g* for 15 min at 4 °C (Biofuge Pico). The resulting supernatant was mixed with ice-cold methanol/water (2:1, 0.5 mL) to facilitate protein precipitation. The samples were then incubated overnight at −4 °C and centrifuged the following day at 17,000×*g* for 15 min at 4 °C (Biofuge Pico).

Quality control (QC) samples were prepared by collecting and pooling 5 µL aliquots from each sample prior to drying overnight in a centrifugal concentrator (SpeedVac). The resulting dried supernatants were then derivatized using the methoximation/silylation protocol provided by Fiehn (2008). Briefly, myristic-d_27_ acid (5 µL of a 6-mM solution in anhydrous pyridine) and U-^13^C-d-Glucose (20 µL of a 1-mM solution in anhydrous pyridine) were added as standards to each sample for retention time locking and quantification purposes, respectively. For the methoximation step, methoxyamine hydrochloride (40 µL of 0.3 M solution in anhydrous pyridine) was added to each sample and the samples were then incubated at 30 °C for 90 min with shaking at 30-min intervals. For the silylation step, the samples were incubated with *N*-methyl-*N*-(trimethylsilyl)-trifluoroacetamide (90 µL; MSTFA) at 37 °C for 30 min. Finally, 2-fluorobiphenyl (10 µL of a 1-mM solution in anhydrous pyridine) was added as an injection standard.

GC–MS analysis was performed on an Agilent 7890 gas chromatograph coupled to a 5975 mass selective detector (MSD) quadruple mass spectrometer (MSD; Agilent Technologies, Santa Clara, California, USA) in accordance with the Fiehn protocol (Fiehn 2008). QC samples were used at the beginning of the run to condition the chromatographic column and thereafter at five sample intervals (Sangster et al. 2006). The acquired spectra were initially processed with the Automated Mass Spectral Deconvolution and Identification System software (AMDIS, NIST, Gaithersburg, Maryland, USA) by using the Fiehnlib library (Kind et al. 2009). The spectra were then transferred to MATLAB (Mathworks), and an in-house developed MATLAB script was then used to manually inspect the chromatographic peaks of the identified metabolites and remove all of the features that were not consistently present in the QC samples, before integration of the remaining features (Behrends et al. 2011).

Overall, the analysis led to the analysis of 38 molecular species which were subsequently tested for statistically significant differences between treatment groups. Reasons for metabolite exclusion prior to analysis included poor chromatographic peak shape, peak overlap and an inconsistent presence in the spectra of QC samples. The 38 selected metabolites were normalized through the fitting of QC-derived polynomial curves for each metabolic feature (Dunn et al. 2011) followed by log median factor normalization to account for inter-batch effects. The overall normalization process was evaluated by principal component analysis (PCA) to explore the effect of each normalization step on the inherent clustering (biochemical similarity) of samples.

### Statistical analysis

Prism 5.0 (Graphpad; La Jolla, CA, USA) was used for nonparametric univariate analysis of the ^1^H-NMR spectroscopic analysis (Kruskal–Wallis with Dunn’s multiple test correction) and for parametric analysis of the log-transformed GC–MS data (analysis of variance (ANOVA) with a Bonferroni multiple correction test). A significance threshold value of *p* < 0.05 was set throughout. Please note that inter-time-point statistical comparisons were avoided due to the fluctuating metabolite levels in the control cohorts over time, such as those of glucose and AMP. Furthermore, six liver samples (*n* = 3 for control at 1 h, *n* = 1 for APAP at 3 h, *n* = 1 for control at 6 h and *n* = 1 for AMAP at 6 h) were excluded from the ^1^H-NMR spectroscopic analysis as they were identified as outliers following a preliminary principal component analysis due to poor spectral quality. Finally, the urine metabolic profiles were not analyzed statistically due to insufficient numbers per treatment group per time-point, as a result of insufficient volume collected from the animals within the short time-collection windows.

## Results

### Liver histopathology

Mice given APAP showed histopathological changes at all time-points examined. At 1 h, one of the treated mice showed a grade 1 centrilobular eosinophilia, while a second animal showed a grade 2 panlobular fat vacuolation (Suppl. Table 1). Two of the APAP-treated animals showed grade 1 focal inflammatory cell infiltration at 1 h post-treatment.

At 3 h, all examined animals showed centrilobular eosinophilia of grades 3–4 and two of these animals with grade 4 centrilobular eosinophilia showed a grade 1 centrilobular necrosis.

**Fig. 1:**
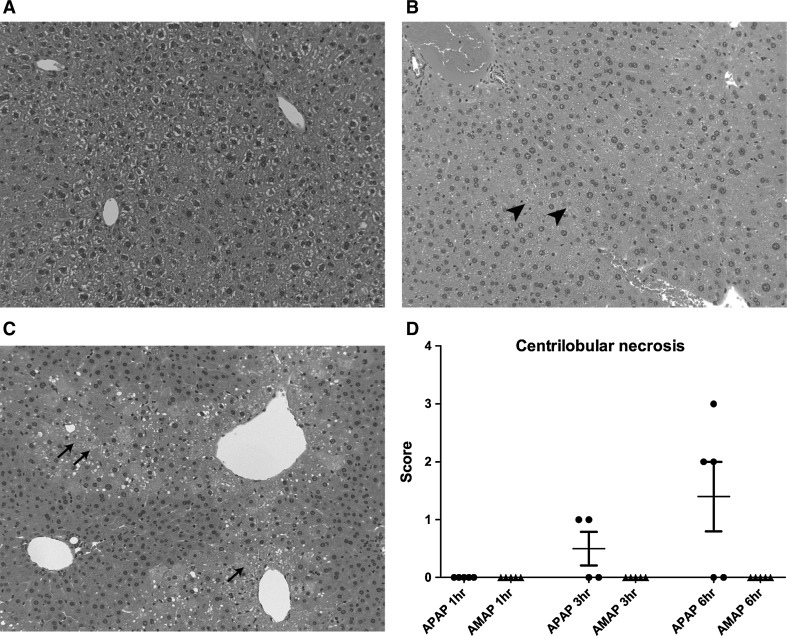
Representative histopathology slides of the APAP-, AMAP-treated and control groups at 6 h. No visible damage is present in the control group (**a**), panlobular fat vacuolation is visible in the AMAP-treated group (**b**; *arrowhead*) and centrilobular necrosis is present in the APAP-treated group (**c**; *arrows*). A summary of the centrilobular necrosis scores of the AMAP- and APAP-treated groups is presented in **d**

At 6 h, four of the five livers examined showed centrilobular eosinophilia of grades 3–4 and three of the five livers examined showed centrilobular necrosis of grades 2–3 (Suppl. Table 1). Figure 1 provides a representative slide of a control (1A), AMAP (1B)- and APAP (1C)-treated animal at 6 h post-treatment showing the presence of the centrilobular necrotic lesion (grade 3) following APAP treatment. A summary of the centrilobular necrosis scores at all time-points in the AMAP- and APAP-treated mice is presented in Fig. 1d.

Mice given AMAP did not show any histological changes at time-point 1 h, but at both time-points 3 h and 6 h the livers of two of five treated mice showed a grade 1 and grade 2 centrilobular eosinophilia while at time-point 6 h one of the mice with centrilobular eosinophilia also showed a grade 2 panlobular fat vacuolation (Suppl. Table 1). Two of the AMAP-treated animals showed a grade 1 focal inflammatory cell infiltration at all time-points, whereas an additional animal showed a grade 1 focal inflammatory cell infiltration at 1 h and 6 h post-treatment (Suppl. Table 1).

### APAP and AMAP metabolism in C57BL/6J mice

**Fig. 2:**
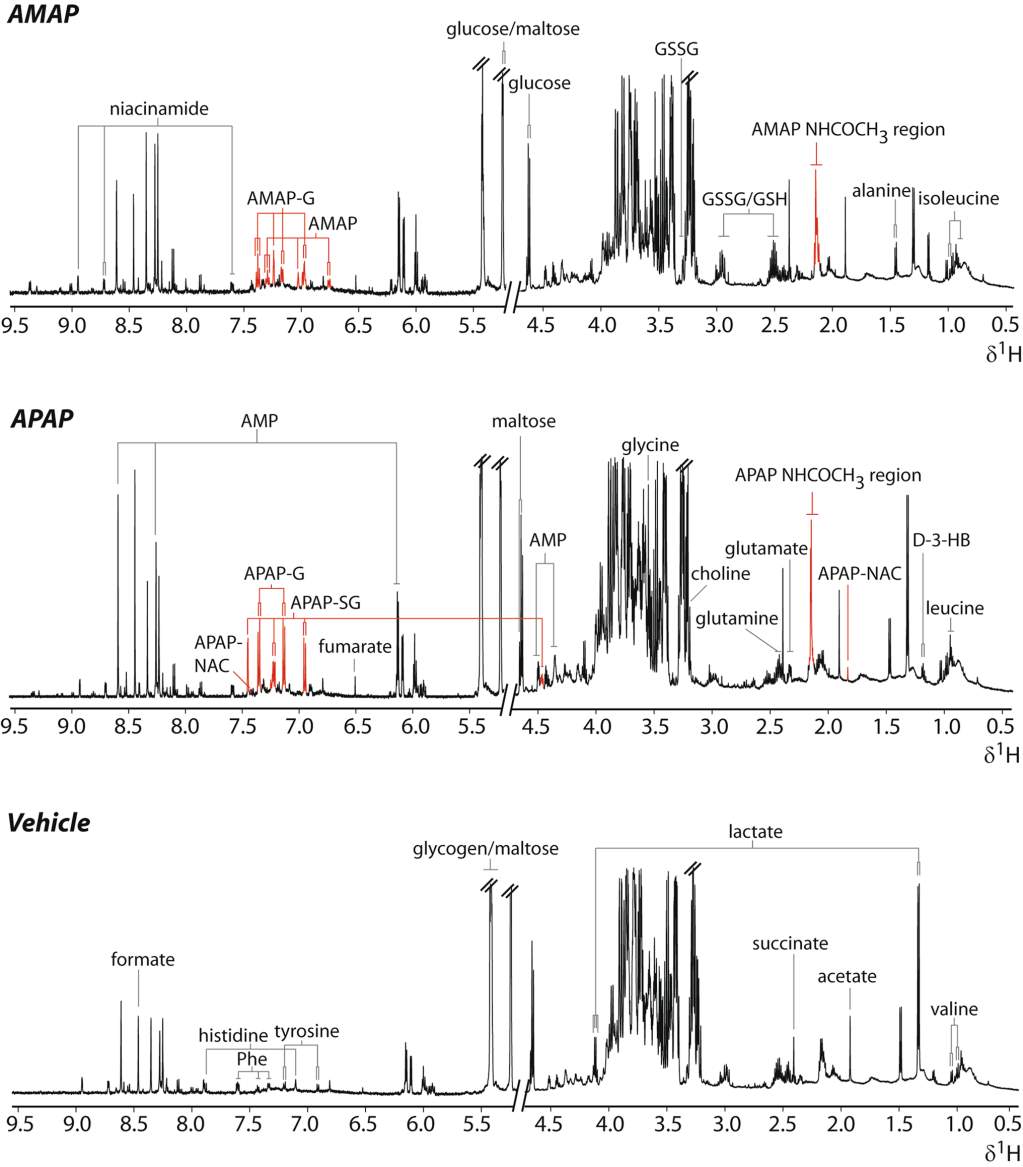
Representative ^1^H-NMR spectra of hepatic extract metabolic profiles of the APAP, AMAP and control groups at 1 h. Resonances assigned to drug-related molecules have been colored in red. Key: APAP/AMAP-G, APAP/AMAP glucuronide; APAP-SG, APAP glutathionyl; APAP-NAC, APAP-*N*-acetylcysteinyl; APAP/AMAP-NHCOCH_3_, APAP/AMAP *N*-acetyl resonance; GSH, reduced glutathione; GSSG, oxidized glutathione; Phe, phenylalanine; d-3-HB, d-3-hydroxybutyrate; AMP, adenosine monophosphate, overlapped resonances from glucose/glycogen/maltose labelled

**Fig. 3:**
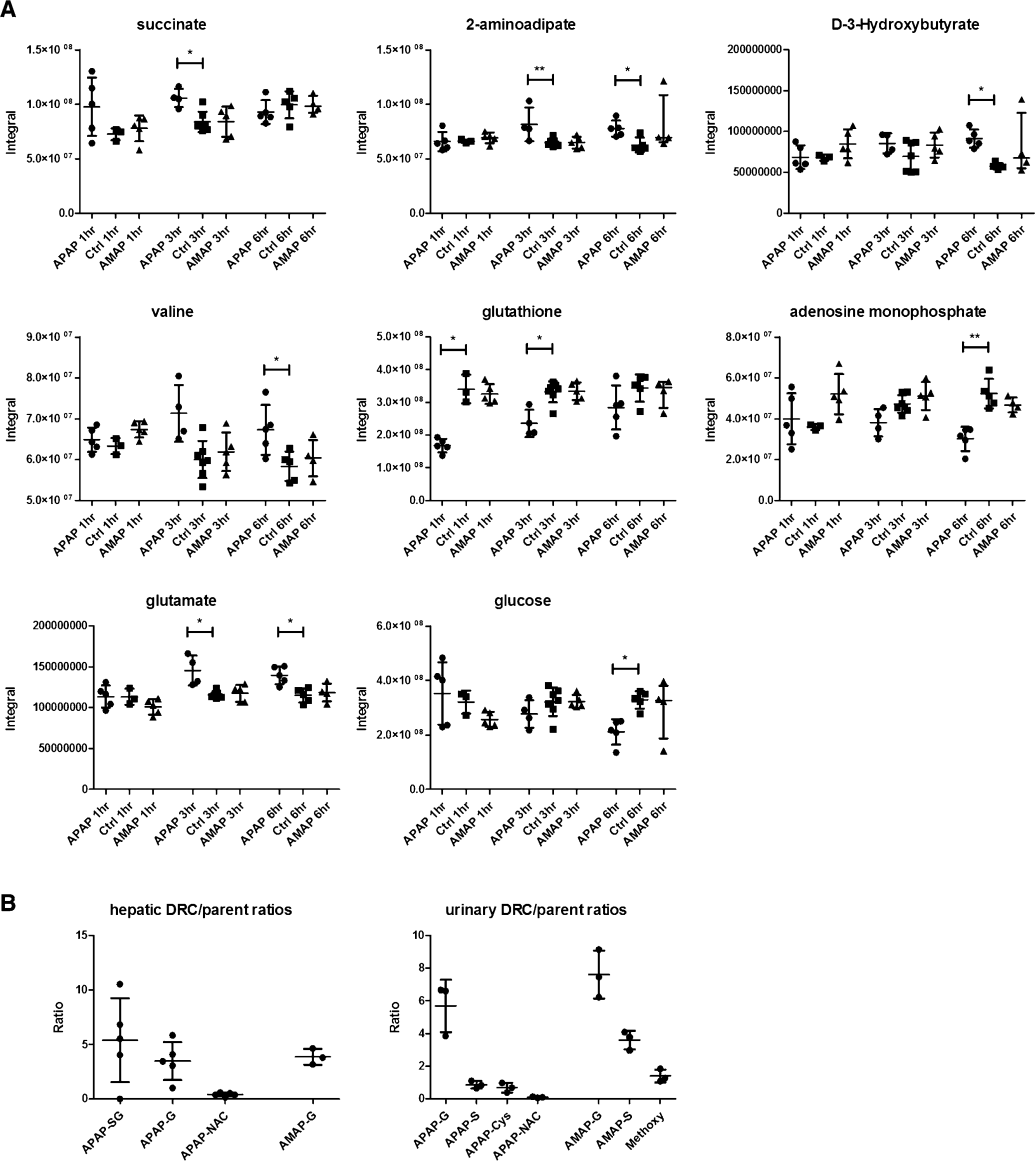
Integrals of discriminatory metabolites identified by the ^1^H-NMR spectroscopic analysis of hepatic extracts after APAP/AMAP administration (**A**). Drug metabolite ratios of APAP and AMAP conjugates to their respective parent molecules in the hepatic extract and urinary metabolic profiles at the 1-h time-point are also shown (**B**). Kruskal–Wallis test coupled to Dunn’s multiple test correction was used to calculate the statistical significance of endogenous metabolic perturbations, which is indicated by the *asterisks*, * and ** (*p* < 0.05 and *p* < 0.01, respectively). *Key* -SG, glutathionyl conjugate; -S, sulfate conjugate; -G, glucuronide conjugate; -Cys, cysteinyl conjugate; -NAC, *N*-acetylcysteinyl conjugate

The metabolism of APAP and AMAP was characterized in hepatic extracts and urine from the 1D ^1^H-NMR spectroscopic profiles. In the hepatic extracts, APAP and its glucuronide, glutathionyl and *N*-acetylcysteinyl conjugates were observed at 1 h (Figs. 2, 3b). In the case of AMAP-treated hepatic extract profiles, the unchanged parent and AMAP glucuronide were the only compounds detected at the equivalent time-point (Figs. 2, 3b). The urinary ^1^H-NMR spectroscopic metabolic profiles revealed the presence of the APAP parent and the APAP glucuronide, sulfate, *N*-acetylcysteinyl and cysteinyl conjugates at 1 h (Suppl. Fig. 1 and Fig. 3b). In the AMAP-treated mice, the unchanged parent and the glucuronide and sulfate conjugates were detected, but there was no evidence for the excretion of glutathionyl-conjugated compounds (Suppl. Fig. 1 and Fig. 3b). The AMAP glucuronide conjugate was the most abundant metabolite in all sample matrices, while the APAP glutathionyl and glucuronide conjugates were the most abundant compounds detected in liver and urine samples from APAP-dosed mice. These data are presented as metabolite/parent ratios for both liver and urine in Fig. 3b. Lower levels of the xenobiotic metabolites were also detected in the urinary metabolic profiles of the APAP (cysteinyl, glucuronide and sulfate conjugates)- and AMAP-treated mice (glucuronide conjugate) at 3 h.

## ^1^H-NMR spectroscopic detected endogenous changes in hepatic extracts following AMAP and APAP administration

Representative ^1^H-NMR spectra of hepatic extracts from each treatment group and a control are given in Fig. 2 showing the respective assignment of the key xenobiotic and endogenous metabolites. Univariate analysis revealed that APAP administration led to the depletion of glutathione (reflecting both oxidized and reduced forms) at 1 h and 3 h, while succinate, valine, 2-aminoadipate and glutamate were elevated at 3 h, relative to controls. At 6 h post-treatment, the APAP-treated mice showed elevation of 2-aminoadipate, glutamate, d-3-HB and valine, while AMP and glucose were depleted relative to controls. In contrast to APAP, AMAP administration did not lead to any statistically significant metabolic differences in the ^1^H-NMR metabolic profiles, relative to controls, throughout the time course of the study. The integrals and summary of the statistically significant differences identified by ^1^H-NMR spectroscopy are presented in Fig. 3a and Table 1, respectively. Table 4 in the Suppl. material displays the individual ^1^H-NMR resonance integral values for all metabolites analyzed.

**Table 1:**
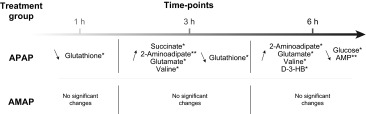
Summary of discriminatory metabolites identified by ^1^H-NMR spectroscopic analysis of hepatic extracts after APAP/AMAP administration

Kruskal–Wallis test coupled to Dunn’s multiple test correction was used to calculate the statistical significance, which is indicated by the asterisks, * and ** (*p* < 0.05 and *p* < 0.01, respectively). Key: d-3-HB—d-3-hydroxybutyrate

### GC–MS-detected endogenous changes in hepatic extracts after AMAP and APAP administration

**Fig. 4:**
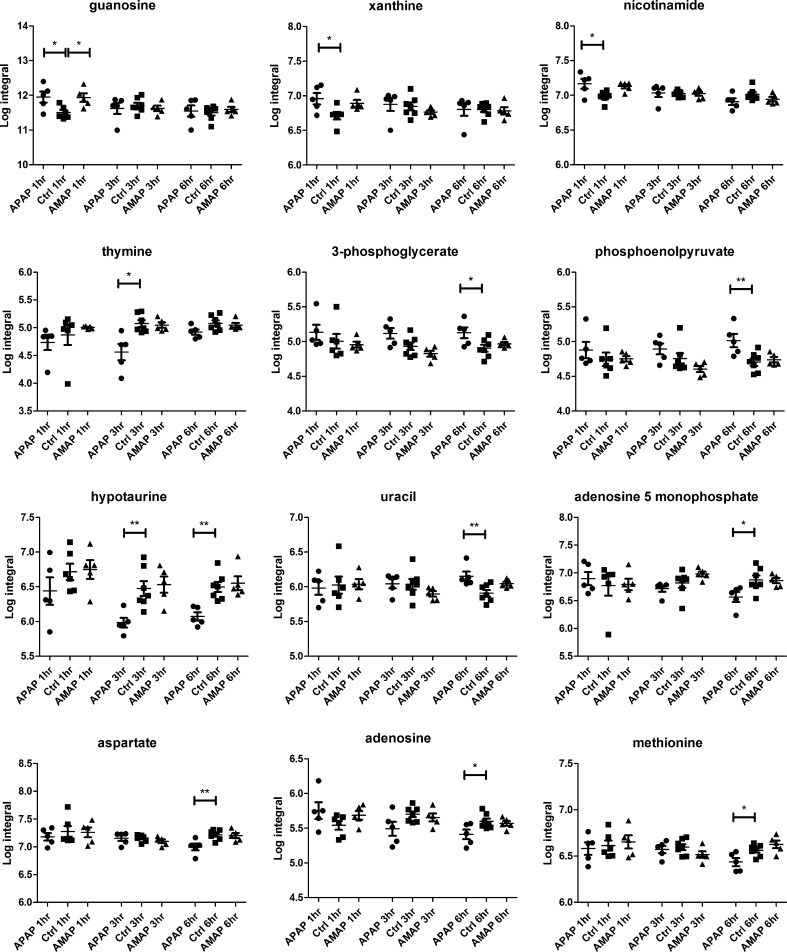
Integrals of discriminatory metabolites identified by GC–MS analysis of the hepatic extracts after APAP/AMAP administration. ANOVA coupled to Bonferroni multiple test correction was used. Statistical significance is shown by the *asterisks*, * and ** (*p* < 0.05 and *p* < 0.01, respectively)

The GC–MS analysis of the hepatic extracts indicated that APAP treatment resulted in elevated quantities of xanthine, guanosine and nicotinamide at 1 h, together with reduced levels of hypotaurine, tryptophan and thymine at 3 h, relative to the controls. At 6 h post-treatment, lower amounts of hypotaurine, adenosine, methionine, AMP, aspartate and tyrosine were observed, while hepatic concentrations of phosphoenolpyruvate, 3-phosphoglycerate and uracil had increased relative to the control mice. The parallel analysis of the AMAP-treated groups revealed elevation of guanosine at 1 h relative to control mice. There were no statistically significant metabolic perturbations at either the 3- or 6-h time-points following AMAP treatment. The integrals and summary of the GC–MS-detected statistically significant hepatic metabolic perturbations induced by both AMAP and APAP are presented in Fig. 4 and summarized in Table 2, respectively. Table 5 in the Suppl. material displays the individual GC–MS integral values for all metabolites analyzed.

**Table 2:**
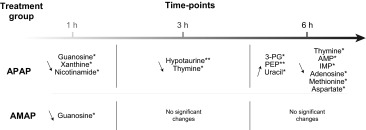
Summary of discriminatory metabolites identified by GC–MS analysis of the hepatic extracts after APAP/AMAP administration

ANOVA coupled to Bonferroni multiple test correction was used. Statistical significance is shown by the asterisks, * and ** (*p* < 0.05 and *p* < 0.01, respectively). Key: IMP—inosine monophosphate; AMP—adenosine monophosphate; PEP—phosphoenolpyruvate; 3-PG—3-phosphoglycerate

## Discussion

**Fig. 5:**
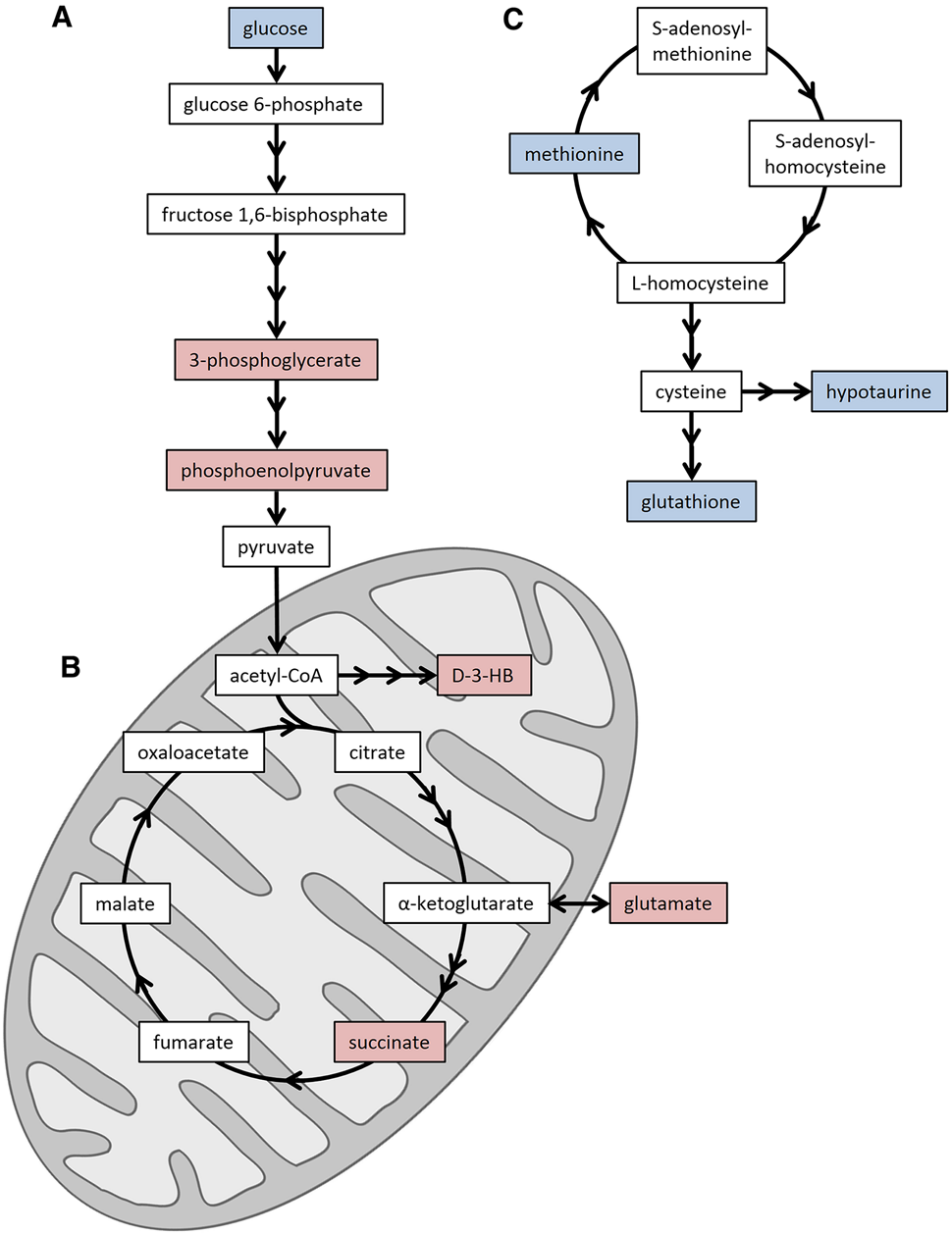
Summary of hepatic metabolic perturbations associated with APAP-induced mitochondrial dysfunction and oxidative stress. Diagrammatic representations of the glycolysis pathway (**a**) and tricarboxylic acid cycle (**b**), as well as the methionine–homocysteine cycle and glutathione synthesis (**c**) are shown. *Red shading* depicts an APAP-induced elevation, while *blue shading* represents an APAP-induced depletion. *Key* d-3-HB, d-3-hydroxybutyrate (color figure online)

The simultaneous characterization of xenobiotic metabolism and endogenous metabolic perturbations induced by AMAP and APAP treatment was achieved using a combination of ^1^H-NMR and GC–MS analysis. This comparative metabonomic approach provided complementary mechanistic insight into the adaptation of hepatic metabolism to toxic insult and enabled the identification of unique metabolic phenotypes for each treatment. Following APAP administration, glutathione conjugates were detected at 1 h post-treatment in the hepatic extracts (APAP glutathionyl, APAP-*N*-acetylcysteinyl) and urinary metabolic profiles (APAP cysteinyl and APAP-*N*-acetylcysteinyl), reflecting both the production and detoxification of the electrophilic reactive metabolite of APAP, NAPQI. This corroborated the marked depletion of hepatic glutathione (sum of reduced and oxidized forms) at 1 h and 3 h post-treatment. This depletion will have likely led to further downstream metabolic effects, such as the GC–MS-detected depletion of hepatic hypotaurine at 3 h and 6 h and methionine at 6 h post-treatment, which are precursors of glutathione. This may reflect the cellular response to APAP-induced oxidative stress which has been previously reported to lead to the depletion of sulfur-containing metabolites such as hypotaurine (Soga et al. 2006; Yamazaki et al. 2013) and methionine (Yamazaki et al. 2013) (Fig. 5).

The observed hepatic elevation of the mitochondrial metabolite succinate, at 3 h post-treatment, as well as glutamate at 3 h and 6 h post-treatment, suggested APAP-induced mitochondrial dysfunction as a key event in the onset of hepatotoxicity. These perturbations which preceded the histopathological observation of necrosis may reflect mitochondrial disruption as a result of the inability of mitochondria to utilize tricarboxylic acid cycle intermediates (Burcham and Harman 1991). APAP administration has been previously demonstrated to up-regulate succinate dehydrogenase (a component of the electron transport chain) in C57BL/6 mice, which would affect succinate levels (Stamper et al. 2011). Finally, disruption of the tricarboxylic acid cycle and the previously reported APAP-induced disruption of glutamate dehydrogenase (Halmes et al. 1996) could also be responsible for the observed elevation of hepatic glutamate.

Impaired mitochondrial respiration and ATP synthesis in response to APAP exposure would lead to the up-regulation of other energy-related pathways (Katyare and Satav 1989). The depletion of glycogen and glucose coupled with the elevation of d-3-HB seen in the hepatic ^1^H-NMR metabolic profiles at 6 h post-treatment provides evidence of the energetic stress exerted by APAP (Coen et al. 2003). This finding suggests a possible shift from mitochondrial oxidative phosphorylation to glycolysis and supports earlier metabonomic-based study findings (Coen et al. 2003, 2004). This shift could also explain the higher levels of hepatic phosphoenolpyruvate and 3-phosphoglycerate that were detected by GC–MS analysis at the same time-point (Fig. 5).

Finally, APAP was also observed to induce a decrease in the hepatic concentration of guanosine at 1 h (GC–MS analysis) and AMP (^1^H-NMR and GC–MS analyses) and adenosine (GC–MS analysis) at 6 h post-treatment. APAP has previously been reported to affect nucleoside and nucleotide metabolism through the inhibition of enzymes such as adenosine deaminase (Ataie et al. 2004); however, the exact mechanism is uncertain. It is possible that the observed hepatic effect could be linked to the depletion of ATP levels and the subsequent utilization of adenine nucleotides in the liver in an attempt to compensate for energy loss (Tirmenstein and Nelson 1990). The panel of APAP-induced discriminatory metabolites and associated metabolic pathways identified in this work is summarized in Fig. 5.

In contrast to APAP, the histopathological analysis suggested that AMAP administration (300 mg/kg) did not lead to hepatotoxicity in the form of centrilobular necrosis within the 6-h timescale studied. The absence of AMAP glutathionyl conjugates in the hepatic and urinary profiles and the absence of perturbation of endogenous hepatic glutathione contrasted with APAP and suggested the absence of AMAP-induced oxidative stress, which could explain the difference in toxicity between the two compounds (Hamilton and Kissinger 1986). This finding is in agreement with previous findings which showed the urinary excretion of the cysteinyl and mercapturic acid conjugates of AMAP represented 5 % of the dose (600 mg/kg, male Swiss Webster mice). In contrast, urinary excretion of the cysteinyl and mercapturic acid conjugates of APAP represented ca. 20 % of the dose (250 mg/kg) (Rashed et al. 1990). Assuming that administration of these isomers at the same doses results in equivalent hepatic exposures, such a result could simply reflect differences in their propensities for bioactivation, or alternatively their relative affinities for alternative routes of metabolism, such as glucuronidation, that result in detoxification.

Following treatment with AMAP, discriminatory metabolites were identified at the earliest 1 h post-treatment time-point with metabolic homeostasis restored at 3 h and 6 h post-treatment, when ^1^H-NMR spectroscopic profiles of AMAP-treated animals were indistinguishable from controls. The histopathology analysis revealed that two animals developed hepatic grade 1 and 2 centrilobular eosinophilia at the 6-h time-point. The presence of centrilobular eosinophilia suggests that AMAP administration may have led to centrilobular necrosis at a later time-point in these two animals. However, within our 6-h equimolar dose comparative study, AMAP was not found to have in common any metabolic perturbations identified in the APAP-treated mice, which included markers for the development of oxidative stress and mitochondrial dysfunction.

In summary, we applied a comparative metabonomic approach to generate metabolic phenotypes of AMAP and APAP exposure, which was anchored with traditional histopathological assessment. APAP administration caused oxidative stress at an early time-point and subsequently induced energetic stress as evidenced from the impairment of mitochondrial function and up-regulation of glycolysis. AMAP was observed to have an early, transient, metabolic effect that was reversible by 6 h and did not lead to a hepatotoxic endpoint.

## Electronic supplementary material

Below is the link to the electronic supplementary material.

Supplementary material 1 (TIFF 704 kb)

Supplementary material 2 (DOCX 71 kb)
