# Supplementary figures and images for: Comparative metabonomic analysis of hepatotoxicity induced by acetaminophen and its less toxic meta-isomer
Source: Arch Toxicol. 2016 Jan 9;90(12):3073–85. doi: 10.1007/s00204-015-1655-x (PMC5104807; doi:10.1007/s00204-015-1655-x)

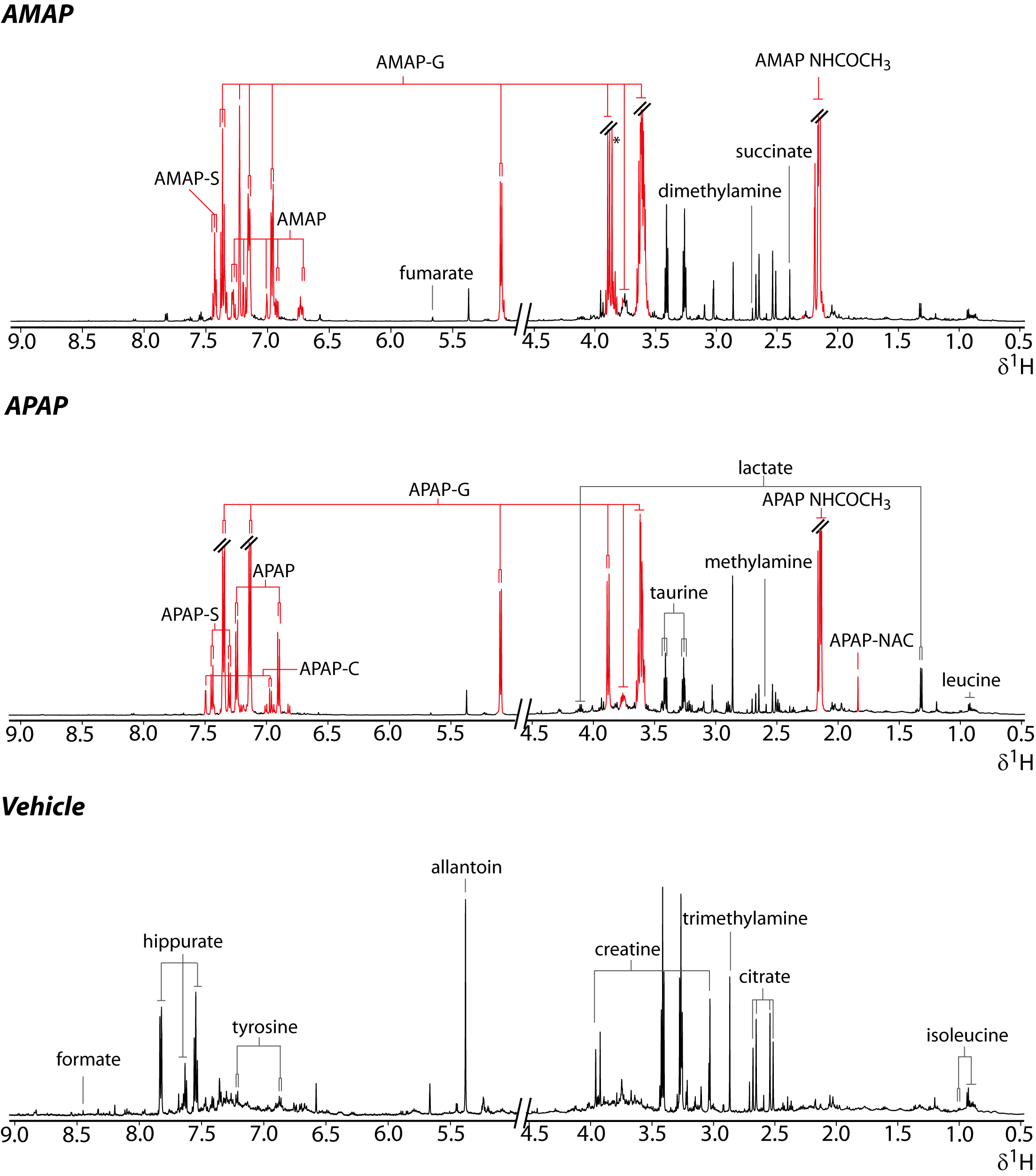

Supplement: Supplementary file 1 — Supplementary material 1 (TIFF 704 kb) [file 204_2015_1655_MOESM1_ESM.tiff]
