# Supplementary material for: Comparative metabonomic analysis of hepatotoxicity induced by acetaminophen and its less toxic meta-isomer
Source: Arch Toxicol. 2016 Jan 9;90(12):3073–85. doi: 10.1007/s00204-015-1655-x (PMC5104807; doi:10.1007/s00204-015-1655-x)
Supplement: Supplementary file 2 — Supplementary material 2 (DOCX 71 kb) [file 204_2015_1655_MOESM2_ESM.docx]

| Histopathology | APAP treated mice | | | | | | | | | | | | | | | | | |  | AMAP treated mice | | | | | | | | | | | | | | | | | | | | |  |
| --- | --- | --- | --- | --- | --- | --- | --- | --- | --- | --- | --- | --- | --- | --- | --- | --- | --- | --- | --- | --- | --- | --- | --- | --- | --- | --- | --- | --- | --- | --- | --- | --- | --- | --- | --- | --- | --- | --- | --- | --- | --- |
|  | ***1 hour*** | | | | |  | | ***3 hours*** | | | |  | ***6 hours*** | | | | | |  | ***1 hour*** | | | | |  | | ***3 hours*** | | | | | |  | | ***6 hours*** | | | | | |  |
| Periportal glycogen | 2 | 1 | 0 | 2 | 2 |  | 1 | | 1 | 0 | 2 |  | 2 | 1 | 0 | 0 | 0 |  | | 2 | 2 | 1 | 1 | 0 | |  | | 2 | 3 | 0 | 2 | 0 | |  | | 2 | 3 | 1 | 1 | 0 | |
|  | | | | | | | | | | | | | | | | | | | | | | | | | | | | | | | | | | | | | | | | | |
| Focal mixed inflammatory reaction | 1 | 0 | 0 | 1 | 0 |  | 0 | | 0 | 0 | 0 |  | 0 | 0 | 0 | 0 | 0 |  | | 0 | 1 | 1 | 0 | 1 | |  | | 0 | 1 | 1 | 0 | 0 | |  | | 0 | 1 | 1 | 0 | 1 | |
|  | | | | | | | | | | | | | | | | | | | | | | | | | | | | | | | | | | | | | | | | | |
| Panlobular fat vacuolation | 0 | 0 | 2 | 0 | 0 |  | 0 | | 0 | 0 | 0 |  | 0 | 0 | 0 | 0 | 0 |  | | 0 | 0 | 0 | 0 | 0 | |  | | 0 | 0 | 0 | 0 | 0 | |  | | 0 | 0 | 0 | 0 | 2 | |
|  | | | | | | | | | | | | | | | | | | | | | | | | | | | | | | | | | | | | | | | | | |
| Centrilobular eosinophilia | 0 | 1 | 0 | 0 | 0 |  | 3 | | 4 | 4 | 4 |  | 4 | 3 | 0 | 3 | 4 |  | | 0 | 0 | 0 | 0 | 0 | |  | | 0 | 0 | 0 | 0 | 0 | |  | | 0 | 0 | 0 | 1 | 2 | |
|  | | | | | | | | | | | | | | | | | | | | | | | | | | | | | | | | | | | | | | | | | |
| Centrilobular necrosis | 0 | 0 | 0 | 0 | 0 |  | 0 | | 1 | 1 | 0 |  | 0 | 2 | 3 | 2 | 0 |  | | 0 | 0 | 0 | 0 | 0 | |  | | 0 | 0 | 0 | 0 | 0 | |  | | 0 | 0 | 0 | 0 | 0 | |

Table 1: Detailed histopathology for AMAP and APAP treated mice

Histology grading

**Glycogen**: grade 1 = vacuolation limited to the cells immediately adjacent to the portal veins; grade 2 = glycogen occupying the periportal region only; grade 3 = glycogen occupying the periportal and midzonal regions; grade 4 = glycogen occupying all three regions of the liver. Control mice can show a variety of gradings as normal variability (relates to dominance in group housed animals).

**Focal inflammatory cell infiltration**: grade 1 = one to two regions of up to 20 mixed inflammatory cells/liver lobe; grade 2 = >2<10 regions of mixed inflammatory cells; grade 3 = 10-20 regions of mixed inflammatory cells; grade 4 = >20 foci of mixed inflammatory cells.

**Panlobular fat vacuolation**: grade 1 = <5% of hepatocytes across the liver lobule showing microvesicular fat vacuolation; grade 2 = 5-10% of hepatocytes across the liver lobule showing microvesicular fat vacuolation. .

**Centrilobular eosinophilia**: grade 1 = <5 cells showing the effect; grade 2 = 5-10 cells showing the effect as a continuous ring of cells around the central vein; grade 3 = 10-20 with 2-3 layers affected around the central vein; grade 4 = all cells affected also including the midzonal regions of the liver lobule. This change is considered to be a precursor of necrosis and is produced by a loss of the basophilic stippling that normal hepatocytes show (mostly due to ribosomes). Affected cells will have normal of sometimes increased nuclear basophilia.

**Centrilobular necrosis**: grade 1 = necrosis of single layer of cells around the central vein affecting <20% of the central veins in the liver lobes; grade 2 = necrosis of single layer of hepatocytes around all of the central veins in the liver lobes; grade 3 = Necrosis of 2-4 layers of hepatocytes round all of the central veins in most liver lobes.

Summary of histopathology:

Mice given AMAP did not show any histological changes at 1hr, but at both t1hr and 6hr the livers of two of five treated mice showed a grade 1 and grade 2 centrilobular eosinophilia respectively while at 6hr one of the mice with centrilobular eosinophilia also showed a grade 2 panlobular fat vacuolation. All other changes recorded in these animals were considered to be normal variability and unrelated to treatment. Mice given APAP showed hepatic changes at all time points examined. At 1hr, one of the treated mice showed a grade 1 centrilobular eosinophilia while a second animal showed a grade 2 panlobular fat vacuolation. At 3hr, all four animals examined showed centrilobular eosinophilia of grades 3-4 and two of these animals showed a grade 1 centrilobular necrosis. At 6hr, four of the five mouse livers examined showed centrilobular eosinophilia of grades 3-4 and three of the five livers examined showed centrilobular necrosis of grades 2-3. All other changes recorded in these animals were considered to be normal variability and unrelated to treatment.

| **Molecule** | **Functional group** | **Integral regions** | | **Multiplicity** |
| --- | --- | --- | --- | --- |
|  |  | Lower | Higher |  |
|  |  |  |  |  |
| **acetate** | CH_3_ | 1.913 | 1.930 | singlet |
| **AMP** | H8 | 8.596 | 8.636 | singlet |
| **2-aminoadipate** | CH_2_ | 2.232 | 2.270 | triplet |
| **D-3-hydroxybutyrate** | CH_3_ | 1.191 | 1.216 | doublet |
| **dimethylamine** | CH_3_ | 2.719 | 2.725 | singlet |
| **formate** | CH | 8.447 | 8.481 | singlet |
| **fumarate** | CH | 6.516 | 6.538 | singlet |
| **glucose** | H1 | 5.234 | 5.268 | doublet |
| **glutamate** | CH_2_ | 2.329 | 2.377 | multiplet |
| **glutathione** | CH_2_ | 2.500 | 2.610 | multiplet |
| **glycine** | CH_2_ | 3.562 | 3.575 | singlet |
| **inosine** | H8 | 8.338 | 8.370 | singlet |
| **lactate** | CH | 4.090 | 4.140 | quartet |
| **niacinamide** | H2 | 8.925 | 8.967 | singlet |
| **succinate** | CH_2_ | 2.398 | 2.416 | singlet |
| **valine** | CH_3_ | 1.031 | 1.058 | doublet |

Table 2: Summary of integrated resonances of endogenous metabolites for ^1^H-NMR statistical analysis

| **Molecule** | **Functional group** | **Integral regions** | | **Multiplicity** |
| --- | --- | --- | --- | --- |
|  |  | Lower | Higher |  |
|  |  |  |  |  |
| ***Hepatic extract*** | | | | |
| **AMAP** | H2 | 7.009 | 7.048 | singlet |
| **AMAP glucuronide** | H2 | 7.227 | 7.258 | singlet |
| **APAP** | H2/H6 | 7.258 | 7.273 | doublet |
| **APAP glucuronide** | H3/H5 | 7.128 | 7.176 | doublet |
| **APAP glutathionyl** | H5 | 6.950 | 6.987 | doublet |
| **APAP N-acetylcysteinyl** | CH_3_ | 1.838 | 1.858 | singlet |
|  | | | | |
| ***Urine*** | | | | |
| **AMAP** | H2 | 7.006 | 7.029 | singlet |
| **AMAP glucuronide** | H2 | 7.221 | 7.259 | singlet |
| **AMAP sulphate** | H5 | 7.405 | 7.460 | triplet |
| **APAP** | H3 | 6.845 | 6.935 | doublet |
| **APAP cysteinyl** | H2 | 7.485 | 7.523 | singlet |
| **APAP glucuronide** | H2/H6 | 7.342 | 7.402 | doublet |
| **APAP N-acetylcysteinyl** | CH_3_ | 1.843 | 1.864 | singlet |
| **APAP sulphate** | H3/H5 | 7.297 | 7.337 | doublet |
| **Methoxy APAP** | CH_3_ | 3.872 | 3.888 | singlet |
|  |  |  |  |  |

Table 3: Summary of integrated resonances of AMAP and APAP related metabolites for ^1^H-NMR statistical analysis

| **Groups** | **Time-point** | **Hepatic extract metabolite integrals by ^1^H-NMR** | | | | | | | | |
| --- | --- | --- | --- | --- | --- | --- | --- | --- | --- | --- |
|  |  |  | | | | | | | | |
|  |  | acetate | AMP | 2-aa | D-3-HB | dimethylamine | formate | fumarate | glucose | glutamate |
|  |  |  |  |  |  |  |  |  |  |  |
| **AMAP** | 1 hour | 70182780 | 37519510 | 61206129 | 61206129 | 5427961 | 61206129 | 61206129 | 61206129 | 61206129 |
|  |  | 78730894 | 37078923 | 67159032 | 67159032 | 6482511 | 67159032 | 67159032 | 67159032 | 67159032 |
|  |  | 69952883 | 35064960 | 63129409 | 63129409 | 5098492 | 63129409 | 63129409 | 63129409 | 63129409 |
|  |  | 83713903 | 45445685 | 68490174 | 68490174 | 6095698 | 68490174 | 68490174 | 68490174 | 68490174 |
|  |  | 94678934 | 46814062 | 64246184 | 64246184 | 5347109 | 64246184 | 64246184 | 64246184 | 64246184 |
|  | 3 hour | 81717855 | 47862426 | 80439026 | 80439026 | 5085105 | 80439026 | 80439026 | 80439026 | 80439026 |
|  |  | 81845316 | 53638059 | 72844593 | 72844593 | 4682977 | 72844593 | 72844593 | 72844593 | 72844593 |
|  |  | 75143864 | 44138006 | 65069693 | 65069693 | 6504000 | 65069693 | 65069693 | 65069693 | 65069693 |
|  |  | 82088707 | 42077865 | 66936397 | 66936397 | 6380810 | 66936397 | 66936397 | 66936397 | 66936397 |
|  |  | 71454128 | 53949666 | 63932998 | 63932998 | 5657090 | 63932998 | 63932998 | 63932998 | 63932998 |
|  | 6 hour | 78576265 | 48093928 | 68429040 | 68429040 | 6698491 | 68429040 | 68429040 | 68429040 | 68429040 |
|  |  | 85945765 | 37365067 | 60142301 | 60142301 | 4214205 | 60142301 | 60142301 | 60142301 | 60142301 |
|  |  | 96499857 | 51915956 | 67414568 | 67414568 | 5670327 | 67414568 | 67414568 | 67414568 | 67414568 |
|  |  | 87603276 | 53548374 | 76475361 | 76475361 | 5779502 | 76475361 | 76475361 | 76475361 | 76475361 |
|  | | | | | | | | | | |
| **APAP** | 1 hour | 81642511 | 36516097 | 63520664 | 63520664 | 7270647 | 63520664 | 63520664 | 63520664 | 63520664 |
|  |  | 80946860 | 25216800 | 65123896 | 65123896 | 5285351 | 65123896 | 65123896 | 65123896 | 65123896 |
|  |  | 78610981 | 49587940 | 61266081 | 61266081 | 7206882 | 61266081 | 61266081 | 61266081 | 61266081 |
|  |  | 60291912 | 33223209 | 53397828 | 53397828 | 5179857 | 53397828 | 53397828 | 53397828 | 53397828 |
|  |  | 69089745 | 56159695 | 59290818 | 59290818 | 6166458 | 59290818 | 59290818 | 59290818 | 59290818 |
|  | 3 hour | 75048898 | 35587455 | 67937150 | 67937150 | 6027401 | 67937150 | 67937150 | 67937150 | 67937150 |
|  |  | 75400845 | 30260292 | 62054930 | 62054930 | 6234966 | 62054930 | 62054930 | 62054930 | 62054930 |
|  |  | 83574139 | 45522037 | 59744383 | 59744383 | 6891697 | 59744383 | 59744383 | 59744383 | 59744383 |
|  |  | 74840627 | 41801416 | 56664486 | 56664486 | 6659195 | 56664486 | 56664486 | 56664486 | 56664486 |
|  | 6 hour | 79165938 | 34944267 | 61073952 | 61073952 | 5838304 | 61073952 | 61073952 | 61073952 | 61073952 |
|  |  | 74142285 | 31844080 | 54362498 | 54362498 | 5502686 | 54362498 | 54362498 | 54362498 | 54362498 |
|  |  | 62280364 | 35279274 | 69448652 | 69448652 | 6383362 | 69448652 | 69448652 | 69448652 | 69448652 |
|  |  | 71244463 | 29557443 | 54991544 | 54991544 | 6019729 | 54991544 | 54991544 | 54991544 | 54991544 |
|  |  | 80122798 | 20815626 | 60017075 | 60017075 | 4798576 | 60017075 | 60017075 | 60017075 | 60017075 |
|  | | | | | | | | | | |
| **Control** | 1 hour | 55977300 | 45304633 | 62787159 | 62787159 | 7039612 | 62787159 | 62787159 | 62787159 | 62787159 |
|  |  | 83341264 | 64217466 | 59514015 | 59514015 | 6732398 | 59514015 | 59514015 | 59514015 | 59514015 |
|  |  | 76596195 | 50871068 | 69298239 | 69298239 | 5844246 | 69298239 | 69298239 | 69298239 | 69298239 |
|  | 3 hour | 91227645 | 49821209 | 66747655 | 66747655 | 5689902 | 66747655 | 66747655 | 66747655 | 66747655 |
|  |  | 76230682 | 67089162 | 69011297 | 69011297 | 7069096 | 69011297 | 69011297 | 69011297 | 69011297 |
|  |  | 79158296 | 53993891 | 67304377 | 67304377 | 5747253 | 67304377 | 67304377 | 67304377 | 67304377 |
|  |  | 59417821 | 42595675 | 64424058 | 64424058 | 6320604 | 64424058 | 64424058 | 64424058 | 64424058 |
|  |  | 69461292 | 51190633 | 61878578 | 61878578 | 6580085 | 61878578 | 61878578 | 61878578 | 61878578 |
|  |  | 80000815 | 53453443 | 59221889 | 59221889 | 6619547 | 59221889 | 59221889 | 59221889 | 59221889 |
|  |  | 86095716 | 60664802 | 63201802 | 63201802 | 6131608 | 63201802 | 63201802 | 63201802 | 63201802 |
|  | 6 hour | 70433273 | 51953210 | 56308256 | 56308256 | 5888876 | 56308256 | 56308256 | 56308256 | 56308256 |
|  |  | 71090666 | 40825880 | 68862698 | 68862698 | 5833210 | 68862698 | 68862698 | 68862698 | 68862698 |
|  |  | 66579413 | 41987012 | 60946046 | 60946046 | 5005524 | 60946046 | 60946046 | 60946046 | 60946046 |
|  |  | 60573983 | 45948158 | 54610347 | 54610347 | 5217563 | 54610347 | 54610347 | 54610347 | 54610347 |
|  |  | 87439796 | 52445686 | 66120403 | 66120403 | 3950361 | 66120403 | 66120403 | 66120403 | 66120403 |
|  |  | 77458297 | 48191371 | 59780131 | 59780131 | 5614167 | 59780131 | 59780131 | 59780131 | 59780131 |

Table 4: Integrals of all hepatic extract metabolites analysed by ^1^H-NMR spectroscopy

| **Groups** | **Time-point** | **Hepatic extract metabolite integrals by ^1^H-NMR** | | | | | | |
| --- | --- | --- | --- | --- | --- | --- | --- | --- |
|  |  |  | | | | | | |
|  |  | glutathione | glycine | inosine | lactate | niacinamide | succinate | valine |
|  |  |  | | | | | | |
| **AMAP** | 1 hour | 342717400 | 61206129 | 30256428 | 61206129 | 12411356 | 61206129 | 61206129 |
|  |  | 297622200 | 67159032 | 44005860 | 67159032 | 11876381 | 67159032 | 67159032 |
|  |  | 370097000 | 63129409 | 31678782 | 63129409 | 8274167 | 63129409 | 63129409 |
|  |  | 303650900 | 68490174 | 19868982 | 68490174 | 8804057 | 68490174 | 68490174 |
|  |  | 312483700 | 64246184 | 26215164 | 64246184 | 10640549 | 64246184 | 64246184 |
|  | 3 hour | 312118500 | 80439026 | 27282181 | 80439026 | 7908554 | 80439026 | 80439026 |
|  |  | 333662900 | 72844593 | 30298395 | 72844593 | 14635484 | 72844593 | 72844593 |
|  |  | 355505300 | 65069693 | 34231828 | 65069693 | 12856604 | 65069693 | 65069693 |
|  |  | 365266500 | 66936397 | 30111592 | 66936397 | 9260516 | 66936397 | 66936397 |
|  |  | 302351800 | 63932998 | 23670533 | 63932998 | 13250466 | 63932998 | 63932998 |
|  | 6 hour | 334596200 | 68429040 | 19762565 | 68429040 | 11106671 | 68429040 | 68429040 |
|  |  | 363722800 | 60142301 | 30676977 | 60142301 | 8960979 | 60142301 | 60142301 |
|  |  | 265998000 | 67414568 | 25192286 | 67414568 | 8200290 | 67414568 | 67414568 |
|  |  | 356712800 | 76475361 | 18748563 | 76475361 | 8763105 | 76475361 | 76475361 |
|  | | | | | | | | |
| **APAP** | 1 hour | 179730600 | 63520664 | 25409867 | 63520664 | 12976012 | 63520664 | 63520664 |
|  |  | 136746200 | 65123896 | 47575963 | 65123896 | 18797800 | 65123896 | 65123896 |
|  |  | 192684400 | 61266081 | 30963971 | 61266081 | 10841613 | 61266081 | 61266081 |
|  |  | 166319400 | 53397828 | 33830646 | 53397828 | 8714124 | 53397828 | 53397828 |
|  |  | 162658800 | 59290818 | 24995457 | 59290818 | 13341620 | 59290818 | 59290818 |
|  | 3 hour | 204452300 | 67937150 | 22167358 | 67937150 | 13662427 | 67937150 | 67937150 |
|  |  | 207159600 | 62054930 | 35918604 | 62054930 | 10502105 | 62054930 | 62054930 |
|  |  | 293514900 | 59744383 | 21516415 | 59744383 | 9316836 | 59744383 | 59744383 |
|  |  | 236367700 | 56664486 | 22868537 | 56664486 | 7699616 | 56664486 | 56664486 |
|  | 6 hour | 296060000 | 61073952 | 22263338 | 61073952 | 8986854 | 61073952 | 61073952 |
|  |  | 301240300 | 54362498 | 21160016 | 54362498 | 12358237 | 54362498 | 54362498 |
|  |  | 331691900 | 69448652 | 34936087 | 69448652 | 7967870 | 69448652 | 69448652 |
|  |  | 387876600 | 54991544 | 37887491 | 54991544 | 13658438 | 54991544 | 54991544 |
|  |  | 362251500 | 60017075 | 21748662 | 60017075 | 6720819 | 60017075 | 60017075 |
|  | | | | | | | | |
| **Control** | 1 hour | 342466200 | 62787159 | 21701123 | 62787159 | 6084904 | 62787159 | 62787159 |
|  |  | 352361700 | 59514015 | 21451180 | 59514015 | 12607039 | 59514015 | 59514015 |
|  |  | 265367800 | 69298239 | 40466056 | 69298239 | 8604979 | 69298239 | 69298239 |
|  | 3 hour | 334936600 | 66747655 | 29482735 | 66747655 | 11652959 | 66747655 | 66747655 |
|  |  | 322185700 | 69011297 | 23088411 | 69011297 | 15221104 | 69011297 | 69011297 |
|  |  | 346472700 | 67304377 | 25334171 | 67304377 | 10866740 | 67304377 | 67304377 |
|  |  | 376039900 | 64424058 | 22835149 | 64424058 | 11141565 | 64424058 | 64424058 |
|  |  | 343336300 | 61878578 | 25820306 | 61878578 | 9852914 | 61878578 | 61878578 |
|  |  | 272970400 | 59221889 | 28055337 | 59221889 | 16300081 | 59221889 | 59221889 |
|  |  | 342677900 | 63201802 | 23526379 | 63201802 | 10149148 | 63201802 | 63201802 |
|  | 6 hour | 353949100 | 56308256 | 30486221 | 56308256 | 12490670 | 56308256 | 56308256 |
|  |  | 373431000 | 68862698 | 28621040 | 68862698 | 11408074 | 68862698 | 68862698 |
|  |  | 342717400 | 60946046 | 28460961 | 60946046 | 11444707 | 60946046 | 60946046 |
|  |  | 297622200 | 54610347 | 21534951 | 54610347 | 10090124 | 54610347 | 54610347 |
|  |  | 370097000 | 66120403 | 22861909 | 66120403 | 8772165 | 66120403 | 66120403 |
|  |  | 303650900 | 59780131 | 19881919 | 59780131 | 8554103 | 59780131 | 59780131 |

Table 4: Integrals of all hepatic extract metabolites analysed by ^1^H-NMR spectroscopy

| **Groups** | **Time-point** | **Log10 hepatic extract metabolite integrals by GC-MS** | | | | | | | | | | |
| --- | --- | --- | --- | --- | --- | --- | --- | --- | --- | --- | --- | --- |
|  |  |  | | | | | | | | | | |
|  |  | adenosine | AMP | aspartate | cholesterol | creatinine | guanosine | hypotaurine | hypoxanthine | IMP | lysine | maltose |
|  |  |  | | | | | | | | | | |
| **AMAP** | 1 hour | 5.637769 | 6.801502 | 7.017809 | 6.872924 | 4.089226 | 12.04149 | 6.809258 | 7.238988 | 6.219318 | 6.259019 | 14.97727 |
|  |  | 5.803157 | 7.153217 | 7.353453 | 6.790837 | 4.293818 | 11.77937 | 6.289221 | 7.165402 | 6.730544 | 6.429650 | 15.05653 |
|  |  | 5.841191 | 6.693954 | 7.483588 | 6.958141 | 4.100870 | 12.33332 | 6.811156 | 7.456311 | 6.251181 | 6.450862 | 15.33360 |
|  |  | 5.481992 | 6.521641 | 7.346098 | 6.815692 | 4.221772 | 11.86672 | 6.708226 | 7.348333 | 6.131701 | 6.500682 | 15.11243 |
|  |  | 5.661234 | 6.791607 | 7.301941 | 6.756753 | 4.138870 | 11.73743 | 7.120798 | 7.269845 | 6.166149 | 6.459989 | 15.08571 |
|  | 3 hour | 5.839199 | 7.104408 | 7.093607 | 6.746838 | 4.323008 | 11.52665 | 6.809856 | 7.089722 | 6.355919 | 6.229479 | 14.92194 |
|  |  | 5.673683 | 6.956243 | 7.142995 | 6.706651 | 4.354951 | 11.64915 | 6.154195 | 6.974615 | 6.636419 | 6.276705 | 14.88306 |
|  |  | 5.685873 | 6.839173 | 7.190235 | 6.776539 | 4.163381 | 11.87839 | 6.579248 | 7.234690 | 6.128008 | 6.432951 | 15.11193 |
|  |  | 5.485606 | 6.973192 | 7.125524 | 6.406851 | 4.327638 | 11.65441 | 6.398103 | 7.037638 | 6.416068 | 6.179956 | 15.09951 |
|  |  | 5.595994 | 7.028903 | 7.048058 | 6.695241 | 4.177763 | 11.36138 | 6.710895 | 7.015835 | 6.491518 | 6.161652 | 14.97863 |
|  | 6 hour | 5.558047 | 6.988736 | 6.990266 | 6.739606 | 4.354604 | 11.41378 | 6.474283 | 7.026533 | 6.428275 | 6.073321 | 15.00167 |
|  |  | 5.619257 | 6.742811 | 7.197686 | 6.805467 | 4.245709 | 11.60202 | 6.936781 | 7.123071 | 6.008478 | 6.305213 | 14.96482 |
|  |  | 5.667489 | 6.880022 | 7.342004 | 6.947313 | 4.305785 | 11.87908 | 6.385220 | 7.168241 | 6.420924 | 6.518633 | 15.12026 |
|  |  | 5.457640 | 6.758873 | 7.126548 | 6.773594 | 4.395130 | 11.51170 | 6.526489 | 7.186734 | 6.422666 | 6.134945 | 14.76558 |
|  |  | 5.548592 | 6.938289 | 7.08869 | 6.749393 | 4.262433 | 11.51268 | 6.437860 | 7.035373 | 6.504909 | 6.165453 | 15.01074 |
|  | | | | | | | | | | | | |
| **APAP** | 1 hour | 5.751682 | 6.777756 | 7.251525 | 6.877565 | 4.315774 | 12.12315 | 6.312535 | 7.226403 | 6.382646 | 6.512164 | 15.14606 |
|  |  | 5.444413 | 6.627127 | 7.192171 | 6.8654100 | 4.000776 | 11.59430 | 6.739084 | 7.217306 | 6.190142 | 6.264903 | 15.00495 |
|  |  | 5.739509 | 6.719466 | 7.340726 | 6.849502 | 4.403344 | 11.99240 | 5.850967 | 7.444313 | 6.030154 | 6.265381 | 15.11491 |
|  |  | 6.184117 | 7.206963 | 6.979370 | 7.110527 | 4.380166 | 12.37059 | 6.298248 | 7.011452 | 6.757887 | 6.544040 | 15.22015 |
|  |  | 5.644366 | 7.155341 | 7.087536 | 6.674960 | 4.277620 | 11.43273 | 6.993441 | 6.999760 | 6.543742 | 6.335282 | 15.00813 |
|  | 3 hour | 5.515857 | 6.746549 | 7.225167 | 6.555752 | 4.187176 | 11.87592 | 5.794850 | 7.155218 | 5.978057 | 6.345065 | 15.19347 |
|  |  | 5.311410 | 6.495036 | 7.228779 | 7.034008 | 4.393153 | 11.69958 | 5.942773 | 7.104974 | 5.572820 | 6.395387 | 15.02870 |
|  |  | 5.230393 | 6.784579 | 6.989861 | 6.822801 | 4.235698 | 10.96999 | 5.998003 | 6.857228 | 6.057028 | 5.917697 | 14.64703 |
|  |  | 5.805247 | 6.764666 | 7.235854 | 6.848026 | 4.365702 | 11.82054 | 6.232910 | 7.196268 | 6.085708 | 6.284822 | 15.02180 |
|  |  | 5.597975 | 6.791732 | 7.088890 | 6.696186 | 4.381856 | 11.68008 | 5.949495 | 7.209277 | 5.940663 | 6.154764 | 14.88633 |
|  | 6 hour | 5.297381 | 6.237336 | 6.982513 | 6.914675 | 4.481934 | 11.00189 | 6.219433 | 7.237701 | 5.289413 | 6.126360 | 14.69913 |
|  |  | 5.215213 | 6.524897 | 6.788814 | 6.828228 | 3.479658 | 11.40735 | 6.203803 | 7.165051 | 5.800602 | 5.334112 | 14.93793 |
|  |  | 5.552559 | 6.729178 | 7.029314 | 6.791821 | 4.288651 | 11.86406 | 5.994520 | 7.088470 | 6.095072 | 6.218661 | 15.00385 |
|  |  | 5.423454 | 6.643871 | 7.164637 | 6.83850 | 4.133464 | 11.58287 | 5.922105 | 7.141966 | 6.017186 | 6.212066 | 14.86576 |
|  |  | 5.567503 | 6.695971 | 7.023762 | 6.893454 | 4.150213 | 11.82677 | 6.024460 | 7.170194 | 5.925370 | 6.033470 | 15.00477 |
|  | | | | | | | | | | | | |
| **Control** | 1 hour | 5.572803 | 6.972895 | 7.150439 | 6.816286 | 4.235330 | 11.49795 | 6.613494 | 7.025142 | 6.300133 | 6.198376 | 14.92760 |
|  |  | 5.660790 | 6.808894 | 7.403937 | 6.685257 | 4.402346 | 11.78748 | 6.979174 | 7.179361 | 6.148925 | 6.470315 | 15.14390 |
|  |  | 5.371021 | 6.968602 | 7.120631 | 6.306970 | 4.208585 | 11.37443 | 6.445735 | 7.003614 | 6.179411 | 6.288173 | 14.94524 |
|  |  | 5.686804 | 7.055512 | 7.114715 | 6.835019 | 4.225450 | 11.62162 | 6.677473 | 7.037220 | 6.563029 | 6.225256 | 15.05757 |
|  |  | 5.331899 | 5.890251 | 7.718125 | 6.851560 | 5.044977 | 11.29951 | 7.141760 | 7.341911 | 5.518240 | 6.032766 | 10.54651 |
|  |  | 5.629328 | 6.927494 | 7.139433 | 6.741021 | 4.234961 | 11.37618 | 6.434211 | 7.033213 | 6.508308 | 6.249052 | 14.94460 |
|  | 3 hour | 5.590454 | 6.899788 | 7.103730 | 6.753073 | 4.433869 | 11.72009 | 6.138411 | 7.116578 | 6.524873 | 6.231641 | 15.00093 |
|  |  | 5.583056 | 6.891557 | 7.158889 | 6.808161 | 4.338180 | 11.58600 | 6.480174 | 7.071312 | 6.316472 | 6.334374 | 14.99712 |
|  |  | 5.609340 | 6.358478 | 7.178288 | 6.910427 | 3.976560 | 11.64930 | 6.305304 | 7.503429 | 5.518209 | 6.220637 | 15.15866 |
|  |  | 5.710773 | 7.060601 | 7.051736 | 6.604447 | 4.211032 | 11.39761 | 6.925097 | 6.943579 | 6.182255 | 6.086019 | 14.85851 |
|  |  | 5.863727 | 6.913645 | 7.205616 | 6.781112 | 4.359029 | 11.99262 | 6.292051 | 7.265578 | 6.365251 | 6.313269 | 15.25143 |
|  |  | 5.770437 | 6.881362 | 7.186814 | 6.776686 | 4.207950 | 11.62401 | 6.803106 | 7.138617 | 6.353469 | 6.291081 | 15.08232 |
|  |  | 5.773112 | 6.724938 | 7.216203 | 6.585586 | 4.304297 | 11.90589 | 6.373639 | 7.277325 | 6.299545 | 6.450708 | 15.16127 |
|  | 6 hour | 5.535671 | 6.955300 | 7.074041 | 6.713666 | 4.287083 | 11.34574 | 6.520365 | 6.961692 | 6.357629 | 6.168180 | 14.78623 |
|  |  | 5.562137 | 6.761769 | 7.264237 | 6.771236 | 4.538445 | 11.59006 | 6.611023 | 7.087099 | 6.447495 | 6.290716 | 15.03932 |
|  |  | 5.590389 | 6.793214 | 7.227701 | 6.881037 | 4.051841 | 11.61320 | 6.378123 | 7.160840 | 6.456142 | 6.148814 | 14.96582 |
|  |  | 5.707775 | 7.177818 | 7.138116 | 6.871635 | 4.366827 | 11.49118 | 6.294966 | 7.087217 | 6.644552 | 6.043124 | 15.01218 |
|  |  | 5.499157 | 6.791631 | 7.191893 | 6.510357 | 4.288513 | 11.07266 | 6.502899 | 6.962475 | 6.280206 | 6.276291 | 14.89884 |
|  |  | 5.781297 | 7.078209 | 7.309934 | 6.731961 | 4.356411 | 11.61291 | 6.842152 | 7.126928 | 6.401283 | 6.441012 | 15.06203 |
|  |  | 5.513178 | 6.539499 | 7.302534 | 6.827135 | 4.448537 | 11.65551 | 6.326822 | 7.238690 | 6.046149 | 6.492422 | 14.96389 |

Table 5: Integrals of all hepatic extract metabolites analysed by GC-MS

| **Groups** | **Time-point** | **Log10 hepatic extract metabolite integrals by GC-MS** | | | | | | | | | |
| --- | --- | --- | --- | --- | --- | --- | --- | --- | --- | --- | --- |
|  |  |  | | | | | | | | | |
|  |  | myo-inositol | methionine | nicotinamide | ornithine | PEP | 3-PG | serine | thymine | uracil | xanthine |
|  | | | | | | | | | | | |
| **AMAP** | 1 hour | 5.774132 | 6.519898 | 7.021168 | 11.98964 | 4.826132 | 4.975303 | 7.089559 | 4.976986 | 6.052094 | 6.845677 |
|  |  | 5.813082 | 6.701028 | 7.173039 | 12.63550 | 4.705386 | 4.916022 | 6.934565 | 5.017010 | 5.824217 | 6.810620 |
|  |  | 5.854101 | 6.883997 | 7.168944 | 12.89596 | 4.856387 | 5.106531 | 7.129866 | 4.985077 | 6.278904 | 7.086581 |
|  |  | 5.625814 | 6.674368 | 7.104881 | 12.38785 | 4.645380 | 4.837396 | 6.918063 | 5.022116 | 6.033439 | 6.859947 |
|  |  | 5.628381 | 6.642549 | 7.173000 | 12.31517 | 4.727648 | 4.928573 | 6.836655 | 5.028193 | 5.997435 | 6.853344 |
|  | 3 hour | 5.755951 | 6.487054 | 7.062489 | 12.17878 | 4.678148 | 4.894201 | 6.824269 | 5.204768 | 5.805926 | 6.718862 |
|  |  | 5.710902 | 6.497796 | 6.957472 | 12.09211 | 4.628817 | 4.841860 | 6.839786 | 4.945047 | 5.803993 | 6.762483 |
|  |  | 5.721654 | 6.638091 | 7.108498 | 12.59004 | 4.705863 | 4.888420 | 6.728833 | 4.976600 | 5.990298 | 6.846608 |
|  |  | 5.809083 | 6.524719 | 7.050449 | 12.27193 | 4.522740 | 4.781077 | 6.941367 | 4.994340 | 5.906201 | 6.793654 |
|  |  | 5.672375 | 6.504245 | 6.942791 | 12.17906 | 4.486641 | 4.655087 | 6.969005 | 5.115854 | 5.980391 | 6.695226 |
|  | 6 hour | 5.742311 | 6.414846 | 6.880395 | 11.78182 | 4.856401 | 5.062968 | 6.873797 | 5.020068 | 6.013402 | 6.643357 |
|  |  | 5.736211 | 6.645545 | 6.996236 | 12.35098 | 4.804674 | 4.974675 | 6.856302 | 4.979418 | 5.982334 | 6.775772 |
|  |  | 5.824413 | 6.736501 | 7.045875 | 12.78597 | 4.680445 | 4.946251 | 7.106561 | 5.010395 | 6.021289 | 6.968508 |
|  |  | 5.783146 | 6.581545 | 6.894265 | 12.40124 | 4.682743 | 4.912652 | 7.306689 | 5.015843 | 6.124175 | 6.787931 |
|  |  | 5.786571 | 6.497381 | 6.906102 | 12.13250 | 4.665850 | 4.876165 | 7.162993 | 5.207321 | 6.088059 | 6.738688 |
|  | | | | | | | | | | | |
| **APAP** | 1 hour | 5.820157 | 6.675336 | 7.097753 | 12.59029 | 4.894948 | 5.022505 | 6.916488 | 4.837511 | 6.086066 | 6.948331 |
|  |  | 5.718056 | 6.636718 | 6.930752 | 12.27547 | 4.760442 | 4.877103 | 7.186711 | 4.835498 | 6.096993 | 6.849305 |
|  |  | 5.950126 | 6.763047 | 7.337812 | 12.36189 | 5.325395 | 5.508301 | 6.780815 | 4.197312 | 6.223800 | 7.154556 |
|  |  | 6.001262 | 6.385193 | 7.221900 | 12.30361 | 4.726966 | 5.121552 | 6.162558 | 4.953949 | 5.801086 | 7.121628 |
|  |  | 5.657022 | 6.479764 | 7.239871 | 12.27878 | 4.689098 | 4.942994 | 6.631917 | 4.849272 | 5.701580 | 6.718119 |
|  | 3 hour | 5.837853 | 6.622397 | 7.125807 | 12.62442 | 4.659126 | 4.977095 | 6.901575 | 4.692655 | 5.985497 | 6.934745 |
|  |  | 5.848966 | 6.600906 | 7.104790 | 12.56065 | 5.091450 | 5.326544 | 6.860427 | 4.381774 | 6.154824 | 6.988223 |
|  |  | 5.615091 | 6.437535 | 6.806282 | 11.65639 | 4.774231 | 4.882638 | 7.056869 | 4.087671 | 5.811849 | 6.500665 |
|  |  | 5.925359 | 6.665507 | 7.029303 | 12.53949 | 4.984443 | 5.123523 | 7.068608 | 4.949003 | 6.123151 | 7.004672 |
|  |  | 5.853136 | 6.529862 | 7.106108 | 12.22436 | 4.965616 | 5.176189 | 6.910939 | 4.696025 | 6.142843 | 6.954938 |
|  | 6 hour | 5.747552 | 6.517167 | 6.775130 | 11.85425 | 5.333003 | 5.364750 | 6.915448 | 4.799304 | 6.413373 | 6.928404 |
|  |  | 5.793061 | 6.334597 | 6.851589 | 10.72330 | 5.096766 | 5.176154 | 6.990542 | 4.935848 | 6.134820 | 6.437829 |
|  |  | 5.710923 | 6.437558 | 6.921271 | 12.15489 | 4.859439 | 4.926304 | 6.863708 | 4.961960 | 6.104894 | 6.855463 |
|  |  | 5.782783 | 6.547186 | 6.935962 | 12.22656 | 4.786940 | 4.941721 | 6.814185 | 4.835395 | 6.044292 | 6.890781 |
|  |  | 5.738350 | 6.339258 | 7.062796 | 11.88641 | 4.996227 | 5.154592 | 6.914659 | 5.076215 | 6.061475 | 6.902726 |
|  | | | | | | | | | | | |
| **Control** | 1 hour | 5.772652 | 6.498319 | 6.993475 | 12.11705 | 4.652038 | 4.801422 | 6.945644 | 4.975355 | 5.863983 | 6.727637 |
|  |  | 5.841611 | 6.706178 | 7.071904 | 12.94619 | 4.753685 | 5.021087 | 7.040188 | 5.047259 | 6.099619 | 6.899190 |
|  |  | 5.710443 | 6.579854 | 7.037603 | 12.38836 | 4.507897 | 4.828292 | 6.970033 | 4.951476 | 5.706244 | 6.707616 |
|  |  | 5.800449 | 6.542653 | 6.961148 | 12.21160 | 4.750338 | 4.965163 | 7.085494 | 5.095601 | 5.908543 | 6.732274 |
|  |  | 6.661741 | 6.839663 | 6.832276 | 11.83968 | 5.193647 | 5.463657 | 7.100236 | 3.987639 | 6.584318 | 6.483351 |
|  |  | 5.767783 | 6.504472 | 6.984740 | 12.12733 | 4.618018 | 4.843970 | 6.961479 | 5.155394 | 5.984276 | 6.731796 |
|  | 3 hour | 5.800422 | 6.496828 | 6.967716 | 12.11002 | 4.626826 | 4.799402 | 6.976400 | 4.916216 | 5.910561 | 6.763310 |
|  |  | 5.721183 | 6.589022 | 6.968791 | 12.37552 | 4.721413 | 4.824869 | 6.786519 | 4.970622 | 5.987349 | 6.749059 |
|  |  | 5.896627 | 6.703033 | 7.052473 | 12.03928 | 5.199632 | 5.166113 | 7.153337 | 4.936794 | 6.398765 | 7.100398 |
|  |  | 5.763305 | 6.492748 | 7.015786 | 12.23063 | 4.615755 | 4.777405 | 6.827608 | 4.992360 | 5.729643 | 6.648491 |
|  |  | 5.852796 | 6.688182 | 7.038642 | 12.44476 | 4.659547 | 4.902514 | 7.207602 | 5.295103 | 6.082239 | 6.883537 |
|  |  | 5.784749 | 6.570084 | 7.047918 | 12.34211 | 4.812050 | 4.996660 | 6.969123 | 5.279246 | 6.028768 | 6.841362 |
|  |  | 5.850946 | 6.635145 | 7.087152 | 12.53503 | 4.646587 | 4.954242 | 6.966041 | 5.137102 | 6.107933 | 6.936102 |
|  | 6 hour | 5.677571 | 6.495457 | 6.972944 | 12.02154 | 4.526941 | 4.712204 | 6.765352 | 5.046066 | 5.738823 | 6.622448 |
|  |  | 5.769234 | 6.592651 | 6.986626 | 12.42269 | 4.808780 | 4.962838 | 6.930026 | 5.007131 | 6.068171 | 6.840526 |
|  |  | 5.823875 | 6.582253 | 6.957857 | 12.18575 | 4.765250 | 5.019344 | 7.079509 | 4.955310 | 6.022295 | 6.885689 |
|  |  | 5.739180 | 6.550573 | 7.016357 | 11.99817 | 4.710624 | 4.847714 | 7.341182 | 5.229013 | 5.806907 | 6.789827 |
|  |  | 5.657100 | 6.464203 | 6.928594 | 11.96076 | 4.545127 | 4.754425 | 6.788123 | 5.141877 | 5.844042 | 6.733949 |
|  |  | 5.786417 | 6.610186 | 7.188930 | 12.53872 | 4.658077 | 4.841992 | 6.823152 | 5.265387 | 5.881184 | 6.844490 |
|  |  | 5.755923 | 6.639204 | 7.052757 | 12.48421 | 4.916390 | 5.063071 | 6.835194 | 4.922601 | 5.994487 | 6.895545 |

Table 5: Integrals of all hepatic extract metabolites analysed by GC-MS
